# The p53 control of apoptosis and proliferation: lessons from *Drosophila*

**DOI:** 10.1007/s10495-014-1035-7

**Published:** 2014-09-13

**Authors:** Bertrand Mollereau, Dali Ma

**Affiliations:** 1 Laboratory of Molecular Biology of the Cell, UMR5239 CNRS/Ecole Normale Supérieure de Lyon, UMS 3444 Biosciences Lyon Gerland, University of Lyon, Lyon, France; 2 Institut de Génomique Fonctionnelle de Lyon (IGFL), Ecole Normale Supérieure de Lyon, CNRS, UMR 5242, University Claude Bernard Lyon-1, 69364 Lyon, France

**Keywords:** p53, Apoptosis, Proliferation, Growth, *Drosophila*

## Abstract

The canonical role of p53 in preserving genome integrity and limiting carcinogenesis has been well established. In the presence of acute DNA-damage, oncogene deregulation and other forms of cellular stress, p53 orchestrates a myriad of pleiotropic processes to repair cellular damages and maintain homeostasis. Beside these well-studied functions of p53, recent studies in *Drosophila* have unraveled intriguing roles of Dmp53 in promoting cell division in apoptosis-induced proliferation, enhancing fitness and proliferation of the winner cell in cell competition and coordinating growth at the organ and organismal level in the presence of stress. In this review, we describe these new functions of Dmp53 and discuss their relevance in the context of carcinogenesis.

## Introduction

The p53 protein is the product of the tumor suppressor gene, TP53. It is a member of the p53 superfamily proteins that comprise TP53, TP63 and TP73. p53 is mutated or inactivated in more than half of human cancers. Early works on p53 have elucidated its canonical function in response to DNA damage. Specifically, in the presence of mild stress or damage signal, p53 blocks cell cycle progression and activates DNA repair machinery to promote cell survival and maintain genome integrity. However, when the damage is extensive, p53 drives cellular senescence or irreversible apoptosis to eliminate the potentially oncogenic cells with genomic instability and therefore reduces the risk of tumorigenesis [1, 2]. In addition, other types of cellular stresses such as starvation and metabolic stress also activate p53 response pathways such as the anti-oxidant response and autophagy [3–6].

**Fig. 1:**
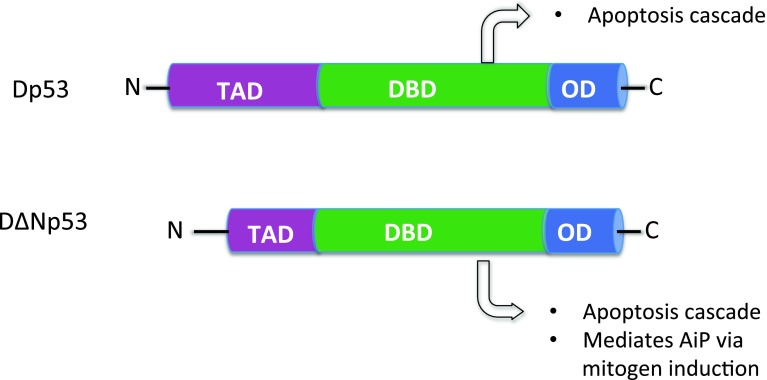
The two isoforms of Dmp53.* TAD*: transactivation domain.* DBD*: DNA binding domain.* OD*: oligomerization domain. DΔNp53 is the predominant fly p53 isoform that activates AiP via the induction of mitogens

The classic model organism *Drosophila* is instrumental to study the pleiotropic functions of p53 in both stress response and development [7]. In 2000, three research groups independently cloned *Drosophila melanogaster* p53 (Dmp53) and found it to be the only member of the p53 family proteins in flies [8–10]. In contrast to all p63-like proteins, Dmp53 lacks a SAM domain. The absence of the SAM domain, along with the initial observation that Dmp53 binds to the reaper (rpr) promoter and activates IR-induced damage response, led the investigators to propose that Dmp53 is a p53 homologue [8–10]. Earlier *Drosophila* studies showed that after irradiation treatment, Dmp53 mediates apoptosis but not cell cycle arrest [10]. Such mode of damage response is reminiscent of that mediated by p63. Therefore, it was proposed that Dmp53 may capture certain features of p63 but has lost its SAM domain during evolution (reviewed in [11]). This is supported by the fact that in some invertebrates such as the *C. elegans,* the p53 homologue, *cep*-*1* still harbors a SAM domain. However, studies from Banerjee and col. have challenged this view as *Drosophila* p53 is able regulate cell cycle upon energy deprivation: the “tenured” mutation disrupts the mitochondrial electron transport chain and causes reduced cellular ATP level, which leads to a G1/S checkpoint arrest that requires Dmp53 [12]. Specifically, reduced ATP activates Dmp53, which in turn promotes the proteasomal degradation of Cyclin E and cell cycle arrest [13]. These results argue that Dmp53, as the vertebrate p53, induces cell cycle arrest, although the mechanistic control and execution have diverged partially during evolution. Thus, the debate about whether Dmp53 acts more like p53 or p63 is still open, rather we favor that as the unique member of p53 family, it is likely *Drosophila* p53 carries the ancestral functions of all p53 family members (Fig. 1).

Since the cloning of Dmp53, studies in the fly primordial germ cells, imaginal discs and adult photoreceptors have not only elucidated the detailed mechanisms underlying *Drosophila* p53-mediated apoptosis, DNA repair and homologous recombination, but also were among the first to assign physiological and developmental functions to p53 [14–19]. Recently, it was shown that Dmp53 is selectively activated in gonadal stem cells subjected to genotoxic or oncogenic stress restricting their growth, thus making the fly reproductive organs an attractive model to study p53 function in stem cells [20].

**Fig. 2:**
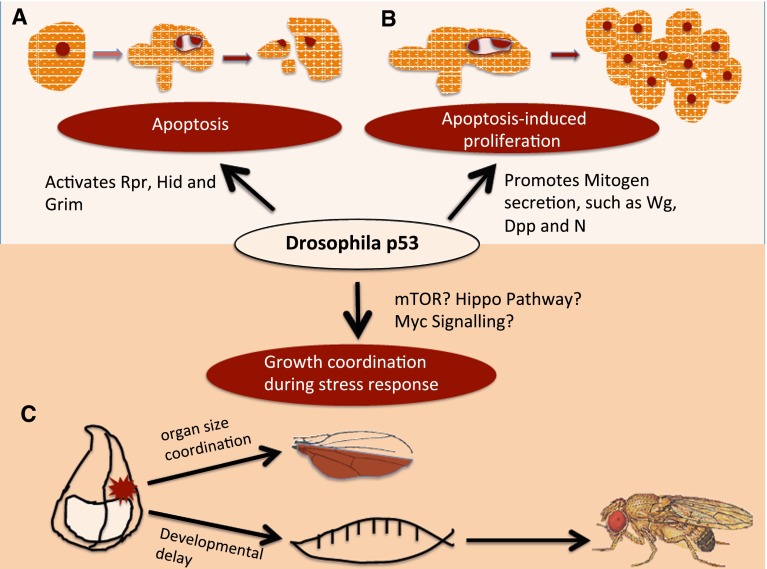
*Drosophila* p53 controls apoptosis, apoptosis-induced proliferation and coordinates organ and systemic growth. (*a*) In the presence of irreparable cellular damage, Dmp53 activates the *Drosophila* pro-apoptotic genes: Rpr, Hid, Grim, which in turn inhibit Diap1 activity to activate the caspase-mediate cell death. (*b*) In the presence of apoptosis, the neighboring cells begin to proliferate to regenerate the damaged tissue. Dmp53 facilitates such apoptosis-induced proliferation by promoting the activation of Wingless (Wg), Decapentaplegic (Dpp) and Notch (N) signaling pathways. (*c*) Recently, it was discovered that Dmp53 can coordinate organ and systemic growth in the presence of cellular stress or tissue injury. For example, after the developing wing discs are subjected to certain level of irradiation or metabolic stress, the growth of the entire wing disc is delayed in a coordinated fashion to form a final well-proportioned adult wing, and the larval growth and pupariation timing is also delayed to allow the tissues and organ to repair. Dmp53 is required in such organ and system growth coordination through a yet unknown mechanism

Beside these well-studied functions of p53, recent studies in *Drosophila* have unraveled intriguing roles of Dmp53 in promoting cell division in apoptosis-induced proliferation, enhancing fitness and proliferation of the winner cell in cell competition and coordinating growth at the organ and organismal level in the context of stress (Fig. 2). In this issue of Apoptosis, Simón et al. reports a novel finding that Dmp53 interacts with Notch in the developing wing and regulates N expression during apoptosis-induced proliferation (AiP) [21]. We therefore focus this review on these new functions of *Drosophila* p53 and discuss their relevance in carcinogenesis.

## Dmp53 regulates apoptosis-induced proliferation (AiP)

In response to damage or injuries in the developing tissue, apoptotic cells promote cell proliferation in the surrounding tissue to repair and regenerate [22, 23]. Apoptotic cells secret mitogens such as Wnt, TGFβ or Hedgehog (homologous to *Drosophila* Wg, Dpp and Hedgehog/Hh, respectively), which in turn induce immediate and local proliferation in the healthy surrounding cells [24–26]. This phenomenon, now defined as the apoptosis-induced proliferation, was first identified and characterized in *Drosophila* [27–29] and is directly controlled by Dmp53 [30]. AiP is distinct from “compensatory proliferation” (CP), which is a complex global injury response that requires coordinated and balanced local proliferation, re-patterning of damaged organs and mediating systemic growth delay at the level of the tissue and the organism. How AiP contributes to CP remains an open question [31].

AiP was first studied using “undead cells”, which are generated by subjecting the cells to a pro-apoptotic signal, such as *rpr* or *hid*, but apoptosis is concomitantly inhibited by the expression of the caspase inhibitor p35 [28, 29]. In this experimental setup, the undead cells constantly activate the apoptotic cascade without undergoing true apoptosis and produce large amount of mitogens that induce hyperplastic growth of the tissue [27, 32]. These studies have first identified the non-apoptotic functions of caspases such as Dronc in promoting mitogen secretion during AiP [33–35]. Importantly, they also uncovered the requirement of Dmp53 and JNK for the production of mitogens [29, 30, 36]. In the absence of Dmp53, the undead cells fail to express Wg and tissue overproliferation is reduced [30]. This particular finding is the first to indicate that besides mediating apoptosis, Dmp53 is also absolutely required for the induction of mitogens and cell proliferation during AiP.

There is evidence that such Dmp53-controlled proliferation and AiP program are conserved throughout evolution. In the planarians, p53 regulates proliferation and self-renewal in adult stem cell lineages [37]. Similarly in mouse studies, p53 activation through the specific inactivation of Mdm2 in the gut, a highly proliferative tissue, leads to apoptosis and proliferation that compensates for cell loss [38]. Furthermore, the AiP program has been shown to promote head regeneration in the *Cnidaria* hydra [22], in that apoptosis is both necessary and sufficient for Wnt3 production [39]. Interestingly, it was also shown that the AiP program is central to the process of tumor repopulation that occurs upon irradiation of cancer cells [40]. In this study, Huang et al. showed that the induction of caspase 3 in irradiated tumor drives the expression of prostaglandin E2, which potently induces proliferation and tumor repopulation in xenograft mouse tumors. As mutations of TP53 is found in 50 % of human cancers, it is an important and interesting question to address if p53 can regulate AiP program in cancerous cells that retain wild-type p53, and if yes, how. Nevertheless, these results indicate that the control of AiP can be therapeutically important in reducing cancer relapse after radiation or chemotherapy.

## Dmp53 regulates AiP through mechanisms involving mitogen activation

What are the molecular underpinnings of the observation that a single Dmp53 gene can regulate two seemingly incompatible processes: apoptosis versus proliferation, in the same cellular context? Part of the answer may arise from the multiplicity of p53 transcriptional targets, which respond differentially depending on stress intensity. It was proposed that in vertebrates, low stress p53 targets promote ROS detoxification, DNA repair while acute stress targets induce apoptosis or senescence [1]. As in vertebrates, *Drosophila* p53 regulates multiple targets essential for apoptosis, DNA repair and proliferation [14, 21, 41]. How does p53 distinguish between these targets upon stress level remains largely unanswered, but a few studies of p53 isoforms provided some clues. In mammals such as mice and humans, p53 expresses up to 12 protein isoforms generated by alternative splicing, codon initiation sites and internal promoters [42, 43]. In *Drosophila,* there are four annotated transcripts for Dmp53, which leads to the expression of two detectable protein isoforms: Dp53 and DΔNp53 (Fig. 1) [41, 44]. Dp53 refers to the full length p53 protein that has an intact transactivation domain and corresponds to the human full-length (TA) p53. DΔNp53 corresponds to the N-terminally truncated human p53 isoforms. Historically, DΔNp53 was the first cloned isoform [8–10]. To distinguish the roles of Dmp53 isoforms in AiP, Dichtel-Danjoy et al. studied the effect of Dp53 and DΔNp53 overexpression in the developing wing imaginal disc [41]. DΔNp53 induced potent expression of Wg and overproliferation, while Dp53 only resulted in weak Wg expression and proliferation. Importantly, the induction of Wg by DΔNp53 also occurred in the absence of both endogenous Dmp53 isoforms, indicating that the DΔNp53 activates its own transcriptional targets needless of any input from the full-length Dp53 isoform. These results indicate that DΔNp53 is the main fly p53 isoform controlling AiP. Thus, each of the *Drosophila* p53 isoforms may assert specific functions to mediate cell death and proliferation in the regenerating tissue. Based on the findings of the overexpression studies, it will be interesting to further dissect the detailed function of each *Drosophila* p53 isoform in AiP using individual Dmp53 mutant isoforms.

In this issue of Apoptosis, Busturia and col. have examined the contribution of Notch (N) signaling to AiP in relation to DΔNp53 [21]. The authors found that ectopic expression of DΔNp53 was sufficient to induce N expression and the proliferation induced by DΔNp53 depends on the level of N expression. The authors first demonstrated that Dmp53 interacts with Notch genetically by studying trans-heterozygotes where removing a copy of wild-type Dmp53 enhances the notched wing phenotype in *N* mutants. Then, by monitoring the expression of a GFP reporter construct under the control of a Notch cis-regulatory element (*Notch*[*2.7*-*NRE*]-GFP) that contains a putative p53 binding site, the authors showed that varying Dmp53 protein levels regulate N gene transcription. This study corroborates previous mouse studies showing that p53 binds *Notch1* promoter and that p53 silencing resulted in reduced Notch expression [45, 46]. The study of crosstalks between Notch and p53 may be very relevant in the growth control of cancer cells [47].

While overexpressing DΔNp53 activates N and Wg expression in the developing wing, it is unclear how N, Wg, Dpp and Hh contribute to the different AiP paradigms. It was proposed that distinct programs of AiP are activated depending on the cellular state [23, 26]. For example, if apoptosis is induced in the proliferating tissues such as the imaginal wing disc or the anterior part of the eye disc, Wg, Dpp and N are activated in a *dronc*- and *dmp53*-dependent manner; while in differentiating tissues such as the posterior part of the eye disc, Hh is preferentially activated in the dying cells via the non-apoptotic activation of the effector caspases Drice and Dcp1 [34]. Thus, distinct apoptotic cascades can induce the AiP program, which in turn activates tissue specific mitogenic signals to promote proliferation and regeneration of the tissue.

An interesting observation is that DΔNp53 induces mitogen expression and proliferation even when apoptosis is inhibited in *dronc* mutants [21, 36, 41]. This led to the proposal that DΔNp53 acts downstream of the apoptotic pathway to induce the secretion of mitogens and cell proliferation [41]. An alternative hypothesis is that DΔNp53 can promote proliferation independently of apoptosis [21, 36]. However, testing the alternative hypothesis is difficult since in virtually all the known experimental setups employed to study the control of AiP by *Drosophila* p53, the induction of proliferation by Dmp53 is prompted by apoptosis upon stress. In the classic DNA damage response, Dmp53 rapidly activates *rpr* expression and hence the apoptosis cascade. A recent study by Shlevkov and Morata further cements the tight association between Dmp53 and the apoptotic cascade by showing that Dmp53 and JNK mutually activate each other and act upstream of the pro-apoptotic genes (rpr, hid, grim) and establish a positive feedback loop that amplifies the initial apoptotic stimuli [48]. Dmp53 activates directly JNK independently of its transcriptional activity, while JNK overactivation induces Dmp53 expression [48–50]. Thus, in the model of Shlevkov and Morata, most stress-induced cell death would be due to the secondary activation of Dmp53 and JNK [48]. Based on these findings, it is an interesting challenge to find a physiological context to support an experiment in which Dmp53-induced proliferation is completely dissociated from the apoptotic machinery.

## AiP in physiological processes?

Another interesting conundrum is why the AiP program is only induced in response to cell injury, but not in developmental apoptosis. In several fly developmental processes, apoptosis is required to reduce the primordial germ cell numbers in the embryo and eliminate the excess neuroblasts in the larva or the supernumerary interommatidial retinal cell during pupal development, and some of these processes are known to depend on Dmp53 [51–54]. Like in the injury response, developmental apoptosis equally engages the classical apoptotic cascade that induces *rpr, hid* and *grim* expression, inhibits DIAP1 and ultimately activates the initiator (dronc) and effector caspases (drice, dcp1) [55], yet such developmental apoptosis has not been observed to induce proliferation in the neighboring cells. What are the molecular and cellular mechanisms that distinguish developmental apoptosis and injury-induced apoptosis? The question is largely unexplored and we can only surmise a few answers. First, the initial signals that trigger the apoptosis during development and upon injury may be quite different. Second, the Dmp53-induced mitogen activation is only observed when AiP is induced following a damage response, thus creating the cellular context for proliferation that is absent in developmental apoptosis. Third, the extent of the activation of each of the apoptotic pathway components is probably different in the injury response versus developmental cell death, which may subsequently impinge on Dmp53 regulation. Therefore, it may be interesting to investigate if and how Dmp53 expression and function can be genetically or epigenetically altered in developmental apoptosis to cull unwanted cells without activation of AiP.

## Role of Dmp53 in coordinating growth at the organ level

In addition to its role in locally controlling AiP as an injury response, Dmp53 also coordinates growth at the organ and possibly systemic level in the presence of genotoxic or environmental stress. In multicellular organisms, growth must be strictly coordinated to form well-proportioned organs that conform to the size of the host. A few years ago, in an elegant study, Mesquita et al. demonstrated that Dmp53 is essential to coordinate growth in the *Drosophila* imaginal wing disc upon growth challenge. Specifically, the authors hampered growth in the posterior compartment of the wing disc, either by the expression of Ricin, a ribosome-inactivating toxin, or by the inhibition of insulin pathway. The cell division and growth rate in the unaffected compartment accordingly slow down to generate an adult wing that is normally proportioned, even though the final size is smaller than the wild-type wing (Fig. 2) [56]. Interestingly, when the authors repeated the same experiments with impaired Dmp53 function in the wing, the final adult wing is no longer well-proportioned, indicating that Dmp53 non-autonomously induces growth delay in the unaffected anterior compartment [56]. Furthermore, the authors showed that only Dmp53, not the Dmp53-controlled apoptotic machinery components, can mediate such long-range growth delay when half of wing is growth-impaired, suggesting that Dmp53 directs a yet unknown mechanism that is independent of apoptosis to coordinate organ growth. This Dmp53-mediated non-autonomous growth delay strikingly contrasts with the results from the previous AiP studies where Dmp53 and caspases promote long-range, non-autonomous proliferation when apoptosis is induced by IR or by the ectopic activation of Rpr or Hid. How can one reconcile these seemingly opposite Dmp53-mediated injury and repair responses? First of all, whether the tissue damage is caused by IR, Ricin poisoning or insulin inhibition, Dmp53-mediated cell death marked by caspase-3 staining is activated autonomously in the damaged tissue. However, unique to the Mesquita et al. study, the cell death caused by Ricin expression or down-regulating insulin response fails to activate mitogens necessary for proliferation, even in the presence of Dmp53 and a fully-operating apoptotic cascade controlled by Dmp53. If one assumes a priori that Dmp53 directly activates mitogens such as Wg, TGF-β and Notch (as published in this issue) to initiate cell proliferation, then why does Dmp53 fail to do so in the study by Mesquita et al.? The answer may simply be that different kinds of injuries lead to different Dmp53 responses. Therefore, Dmp53 seems to possess the intrinsic capacity to distinguish damage of different nature, i.e. caused by massive DNA damage (IR) or metabolic stress (InR impairment). As mentioned above, the two different Dmp53 isoforms have different capacity to mediate AiP [41]. Based on this knowledge, it is intriguing to postulate that the different Dmp53 isoforms are activated by different kind of cellular stress, so that one isoform predominantly promotes proliferation and the other coordinates growth delay depending on the stress context. Lastly, by overexpression of PTEN or Ricin, the mTOR pathway function is compromised; in mammalian studies, p53 is both an upstream inhibitor and downstream target of the mTOR signalling, which controls cellular and systemic response during development and stress [57]. Therefore, it will be interesting to see if the long-range growth delay mediated by Dmp53 impinges on the mTOR pathway in an isoform-dependent fashion.

In addition to coordinating growth at the organ level, Dmp53 participates to control systemic growth of the organism in response to damage. If a developing organism is subjected to severe injury, such as diffuse irradiation, the overall development is delayed to enable organ repair and regeneration [58]. One of the best demonstrations of this principle uses *Drosophila* wing as a regenerative model. For example, after irradiation, wild-type fly larvae undergo developmental delay, but emerge with normal-looking organs such as the wing. In a recent study, Wells and Johnston found that after general irradiation, *Drosophila* Dmp53 mutant larvae fail to delay developmental timing, and adults emerge with apparent missing tissues on the wing [36]. This observation confirms the idea that the local Dmp53-mediated AiP and the system-wide CP response need to be coordinated during development. How Dmp53 mediates the coordination between organ regeneration and system growth is an open question. Recent studies show that Dilp8 is responsible for coordinating organ growth and developmental timing [59]. It will be interesting to test whether Dmp53 genetically interacts with Dilp8 in such process.

## Role of Dmp53 in cell competition

Cell competition is a cellular phenomenon that occurs when two populations of cells of different “fitness” encounter, and the cells with “fitness advantages”, termed “winner cells”, rapidly expand at the expense of the less fit cells, termed “loser cells” [60]. A widely used model of cell competition is established in *Drosophila* wing discs where a population of Myc-overexpressing cells can outcompete their neighboring wild-type cells that are eliminated by apoptosis [61, 62]. In a recent study by de la Cova et al., Dmp53 is shown to be required for the dMyc overexpressing cells in the wing disc to achieve such “supercompetitor” status through metabolic reprogramming [63]. Specifically, overexpression of Myc increases the glycolytic flux and activates Dmp53 expression. Interestingly, the authors demonstrated that Dmp53 asserts different metabolic control depending on the cellular context: in non-competing cells that uniformly express dMyc, dmp53 activation promotes oxidative phosphorylation to counteract the dMyc-induced glycotyic flux. In this context, Dmp53 asserts its canonical role of the tumor suppressor, in that it responds to oncogene deregulation and acts to minimize the potentially deleterious effect of the activated oncogene, which in this case, is mediated by Myc. However, de la Cova et al. further demonstrate that in a heterogeneous population of cells where dMyc is selectively activated, Dmp53 is required for the increased glycolytic flux in the Myc-overexpressing cells, which is an indispensible step for the Myc-expressing cells to become the “winner cells”. Therefore, during cell competition, Dmp53 acts as an accomplice of Myc to aid in realizing its oncogenic potential. As cell competition is a predominant feature in cancer cell expansion at the detriment of the neighboring normal cells, this study furnishes disconcerting evidence that reveals the cooperation between an oncogene and a tumor suppressor gene to confer the winning status to cancer cells.

## Dmp53 and the Hippo pathway

The Hippo signaling cascade is one of the most conserved organ size control pathways in evolution [64–66]. By phosphorylating Yorkie, the main downstream output the Hippo pathway, the Hippo kinase complexes limit cellular proliferation and permit apoptosis during cellular and tissue growth [64–66]. Almost 10 years ago, a study by Colombani et al. first demonstrated that in the presence of IR damage, Dmp53 acts upstream of the Hippo pathway to induce apoptosis [67]. Specifically, the authors first induced apoptosis by irradiation or tissue-specific overexpression of Dmp53 in the fly imaginal discs and found that caspase-3 level is markedly reduced in mutant clones of Hippo pathway components, suggesting that Hippo acts downstream of Dmp53 to elicit cellular and tissue-wide apoptotic response. Furthermore, in Kc cell lysate and extracts from the *Drosophila* ovaries, IR treatment rapidly leads to phosphorylation of Hippo at a threonine residue (T195), and reducing or removing Dmp53 drastically diminishes such Hippo phosphorylation, and consequently dampens Hippo pathway activation in the presence a cell-death signal. This work nicely demonstrates that Hippo acts epistatically to Dmp53 to control the activation of apoptosis. Now in the light of the finding that Dmp53 can coordinate organ size and systemic growth in a stress context, an intriguing question begs the answer: can Dmp53 act in concert with the components of the Hippo pathway to control and coordinate tissue growth? And vice versa, does the Hippo pathway play a role in AiP in the classic IR-induced damage model? We do not yet have answers. However, the analysis of *yorkie* function may hold some clues. It has been well-established that Yorkie activates Diap1 to limit apoptosis during tissue proliferation and growth [68]. Recently, a study from Zhang and Cohen added the role of Dmp53 to the picture: they first found that Yorkie negatively regulates the expression of Rpr [69]. Reducing Yorkie level in the fly inter-vein wing region leads to specific undergrowth, presumably due to the over-activation of Rpr, and such undergrowth can be partially rescued by the concomitant expression of a dominant negative Dmp53 in the same wing region. Presumably, Yorkie knockdown destabilizes Diap1, hence activates Rpr and the subsequent Dronc-Dmp53 feedback loop [48, 68]. However, the authors observed that in their particular experimental setting, the observed Rpr and Dmp53 activation is independent of the feedback loop, but a result from inferred physical binding between the *Drosophila* ASPP1 homologue and Dmp53, which can activate the downstream apoptotic genes [70, 71]. The authors do not further demonstrate how and to what extend Dmp53 is activated by Yorkie down-regulation, therefore the results can have different implications. One possibility is that physiological Yorkie activity is required to keep Dmp53 level in check to prevent ectopic apoptosis during development. Another interesting possibility is that, despite its seeming inactivity, Dmp53 may act as a sensor of the Hippo pathway status through Yorkie. For example, one may postulate that in actively dividing tissue like the wing disc, dampening Yorkie activity compromises the normal cellular growth potential, hence reduces the “fitness” of the cells. As Dmp53 can act as a sensor for cellular “fitness” as mentioned in the previous section, Yorkie knockdown can lead to enhanced Dmp53 activity to promote apoptosis. These two mechanisms are not necessarily mutually exclusive. Furthermore, can such proposed Dmp53-Yorkie interaction coordinate the growth of a developing organ? The answers can only come from more detailed study of Dmp53 interaction with the Hippo pathway components.

## Conclusions

Studies of p53 in the past 35 years have depicted the many facets of p53 functions that are forever evolving. We now recognize that besides being the classic “guardian of the genome” [72], p53 participates in the regulation of complex physiological and developmental processes [43]. With only one p53 family member expressed as two detectable isoforms, *Drosophila* is a genetically tractable model to explore the “primordial” functions of p53 in addition to its well-known functions in mediating apoptosis in the presence of genotoxic stress. In fact, even such canonical Dmp53 function is more complex than we first realized, as studies begin to unravel the roles of Dmp53 isoforms in controlling AiP and programmed cell death at the same time in the same tissue. The molecular details of these processes require future investigation. Furthermore, studies in *Drosophila* pioneered many founding works in identifying the physiological functions of p53, including the recent discoveries of the role of Dmp53 in controlling organ size, system growth and cell competition in the presence of stress. From an evolutionary point of view, is this kind of growth-control function part of the “primordial” p53 functions? Four years ago, a study from John Abram’s laboratory discovered that Dmp53 mediates meiotic recombination in the germ-line, and proposed that such primordial function of p53 is the origin of the genotoxic stress response [19]. Now an interesting question is whether the AiP mediated by Dmp53 precedes or succeeds the growth control function of Dmp53 during evolution. The answer will certainly be complex and unexpected, as much as it has been in the past 35 years.
